# The characteristics and expression profiles of the mitochondrial genome for the Mediterranean species of the *Bemisia tabaci* complex

**DOI:** 10.1186/1471-2164-14-401

**Published:** 2013-06-17

**Authors:** Hua-Ling Wang, Jiao Yang, Laura M Boykin, Qiong-Yi Zhao, Qian Li, Xiao-Wei Wang, Shu-Sheng Liu

**Affiliations:** 1 Ministry of Agriculture Key Laboratory of Agricultural Entomology, Institute of Insect Sciences, Zhejiang University, Hangzhou, 310058, China; 2 ARC Centre of Excellence, Plant Energy Biology, The University of Western Australia, 35 Stirling Highway, Crawley, 6009, Australia; 3 The University of Queensland, Queensland Brain Institute, Brisbane, Qld, 4072, Australia

**Keywords:** *Bemisia tabaci*, Gene expression, Mediterranean, Mitochondrial genome, Mitogenomics, Whitefly

## Abstract

**Background:**

The whiteflies under the name *Bemisia tabaci* (Gennadius) (Aleyrodidae: Hemiptera) are species complex of at least 31 cryptic species some of which are globally invasive agricultural pests. Previously, the mitochondrial genome (mitogenome) of the indigenous New World *B. tabaci* species was sequenced and major differences of gene order from the postulated whitefly ancestral gene order were found. However, the sequence and gene order of mitogenomes in other *B. tabaci* species are unknown. In addition, the sequence divergences and gene expression profiles of mitogenomes in the *B. tabaci* species complex remain completely unexplored.

**Results:**

In this study, we obtained the complete mitogenome (15,632 bp) of the invasive Mediterranean (MED), which has been identified as the type species of the *B. tabaci* complex. It encodes 37 genes, including 13 protein-coding genes (PCGs), 2 ribosomal RNAs and 22 transfer RNAs (tRNA). Comparative analyses of the mitogenomes from MED and New World (previously published) species reveal that there are no gene arrangements. Based on the Illumina sequencing data, the gene expression profile of the MED mitogenome was analyzed. We found that a number of genes were polyadenylated and the partial stop codons in *cox1*, *cox2* and *nd5* are completed via polyadenylation that changed T to the TAA stop codon. In addition, combining the transcriptome with the sequence alignment data, the possible termination site of some PCGs were defined. Our analyses also revealed that *atp6* and *atp8*, *nd4* and *nd4l*, *nd6* and *cytb* were found on the same cistronic transcripts, whereas the other mature mitochondrial transcripts were monocistronic. Furthermore, RT-PCR analyses of the mitochondrial PCGs expression in different developmental stages revealed that the expression level of individual mitochondrial genes varied in each developmental stage of nymph, pupa and adult. Interestingly, mRNA levels showed significant differences among genes located in the same transcription unit suggesting that mitochondrial mRNA abundance is heavily modulated by post-transcriptional regulation.

**Conclusions:**

This work provides novel insights into the mitogenome evolution of *B. tabaci* species and demonstrates that utilizing RNA-seq data to obtain the mitogenome and analyze mitochondrial gene expression characteristics is practical.

## Background

*Bemisia tabaci*, the sweet potato whitefly, causes millions of dollars of crop damage globally [1,2] and is considered one of the world’s top 100 invasive species according to the International Union for the Conservation of Nature and Natural Resources (IUCN) (http://www.issg.org). It is capable of causing extensive damage to major vegetable, grain legume and fiber crops and regarded as a regulated species by a number of countries or regions, e.g., Australia, Africa, China, the EU, and the USA. There are two main types of damage caused by *B. tabaci*; the first is caused by immature and adult stages feeding (they ingest phloem sap and this causes damage). The second type is indirect damage from excretion of honeydew onto the surfaces of leaves and fruit and this promotes the growth of sooty mold fungi which uses honey dew as a substrate and colonizes contaminated surfaces, further interfering with photosynthesis, ultimately resulting in reduced quality of fruit and fiber [3]. In addition, *B. tabaci* is the vector of many economically important plant viral-pathogens, most being begomoviruses (Geminiviridae); a group recognized as the most important emerging plant virus group in subtropical and tropical world regions [4-6].

*B. tabaci* is now known as a species complex with dozens of morphologically indistinguishable species and contains both invasive and native members [7-10]. In 2007, the first global mitochondrial cytochrome oxidase I (mtCOI) dataset for *B. tabaci* was used to reconstruct the global phylogenetic relationships, indicating significant variation between and within genetic groups [11]. Since that pivotal work in 2007, *B. tabaci* has been shown to be a species complex with at least 31 distinct genetic groups identified based on mtCOI [7,8,10-12]. What is more, by matching museum syntypes from the 1889 original specimen from Gennadius using mtCOI molecular maker, MED were recognized as the type species of the *B. tabaci* complex [13]*.* With the taxonomy of the *B. tabaci* species complex becoming clearer, it is now possible to use this information to carry out detailed comparative studies. This includes uncovering and comparing the mitochondrial genomes (mitogenomes) of the different species in the *B. tabaci* complex. Thao et al. (2004) sequenced the mitogenomes of six whitefly species and found that four of them had an rearrangement of the *cox3 - nd3* region compared to the hypothesized ancestral insect mitochondrial gene order [14]. They suggest, based on this rearrangement that this region has been transposed, at least four times in the evolution of whiteflies. However, whether the gene order of mitogenome in other *B. tabaci* species, especially the invasive MED and MEAM1, differs from the ancestral whitefly mitogenome is still unknown. Furthermore, in the *B. tabaci* species complex, only the mitogenome of New World species is available [14], a detailed comparison of mitogenomes between members of the *B. tabaci* species complex is still lacking. In this study, we decided to explore variation in the mitogenomes of the *B. tabaci* species complex. This study will also add to the growing literature on insect comparative mitogenomics of closely related species [15-20].

In addition to using the mitogenome for evolutionary study, we are also interested in the function of the mitochondrial genes. The mitochondrion is an important organelle responsible for numerous important cellular functions in insects such as energy transduction, apoptosis, detoxification, signal transduction and ATP production [21,22]. With few exceptions, insect mitogenomes contain 37 genes encoding 13 protein coding genes (PCGs), 2 ribosomal RNAs (*rrnL* and *rrnS*) and 22 transfer RNAs (tRNAs) [24]. The gene organization of mitogenomes is different between insects [25,26] and mechanisms of mitochondrial gene expression have been investigated in various organisms [27-29]. Studying the mitochondrial gene organization and expression may facilitate the elucidation of mitogenome evolution and characterization of key components regulating insect biology [23]. At present, large amount of information about mitochondrial expression profile has focused on studies of humans, mice or *Drosophila*[30]. Relatively little is known about the features of non-model insect mitogenomes, such as polyadenylation and modes of gene transcription [28,31-33].

Next-generation sequencing (NGS) data is important to predict processed mitochondrial transcripts and reveal transcription process in mitogenomes [21,34,35]. For example, the transcription profile of genes encoded in the mitogenome of *Drosophila* and the legume pod borer *Maruca vitrata* has been revealed using NGS data [21,34]. For *B. tabaci*, the transcriptome of MED has been sequenced and a total of 43 million reads were obtained [36]. A large number of sequencing reads could be mapped to the mitogenome of the New World *B. tabaci* species (data not shown). Therefore, a second goal of this study is to utilize NGS data to analyzing the characteristics of whitefly mitochondrial gene expression, including the translation start site of PCGs, polyadenylation and polycistronic transcripts.

## Results

### Obtaining the MED mitogenome sequences with the NGS data

Without prior amplification of specific regions of the mitogenome, DNA sequence data obtained by NGS methodology can generate sequences of the mitogenome [35]. In this study, 43 million Illumina sequencing reads of MED [36] were used to retrieve the MED mitogenome sequences by mapping them to the available mitogenome of *B. tabaci* New World species (GenBank accession number: AY521259). A total of 635,172 reads were mapped to the New World species reference mitogenome. These reads were assembled into eight contigs distributed in different parts of the New World mitogenome (Additional file 1). The missing base positions were 1–104, 624–800, 2042–2211, 2237–2282, 7523–7595, 11209–11434, 11537–11614 and 13566–13762. Specific primers were then designed to close these gaps (Additional file 2).

### The MED mitogenome

**Figure 1:**
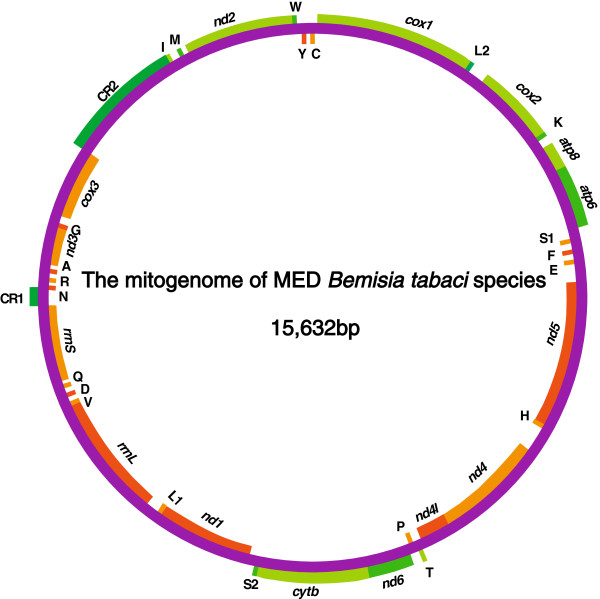
**The mitogenome of the MED species.** Genes coded in the N strand (clockwise orientation) are pine and cypress green or bean green colored. Genes coded in the J strand (anti-clockwise orientation) are tangerine or bright yellow colored. CR1, CR2 represent control regions. tRNA-one letter amino acid abbreviation (parenthesis three letter amino acid abbreviation followed by anticodons): tRNA-A (ala), tRNA-C (cys), tRNA-D (asp), tRNA-E (glu), tRNA-F (phe), tRNA-G (gly), tRNA-H (his), tRNA-I (ile); tRNA-K (lys), tRNA-L1 (leu), tRNA-L2 (leu), tRNA-M (met), tRNA-N (asn), tRNA-P (pro), tRNA-Q (gln), tRNA-R (arg), tRNA-S1 (ser), tRNA-S2 (ser), tRNA-T (thr), tRNA-V (val), tRNA-W (trp), and tRNA-Y (tyr). CR1: the first putative control region; CR2: the second putative control region.

The complete mitogenome of MED is 15,632 bp and encodes the 37 genes (13 PCGs, 22 tRNA and 2 rRNA genes), found in most metazoan mitogenomes (Figure 1 and Table 1) [37]. The MED mitogenome also includes 10 non-coding portions (intergenic spacers), which are at least 10 bps long. Two pairs of genes (*atp6* and *atp8*; *nd4* and *nd4l*) are located on the same strand and overlap by 10 bp and 3 bp, respectively, which is a common feature of insect mitochondrial DNA [31,32]. The other genes are contiguous or separated by a few nucleotides or intergenic spacers (Figure 1). The entire complement of 22 tRNAs ranging in size from 63 to 78 bp was found and most of the tRNAs can be folded as classic clover-leaf structures except tRNA-SerUCU that is missing the dihydorouridine (DHU) arm as in most metazoan. Compared to the New World mitogenome (15,332 bp), there is a slight expansion of MED (15,632 bp) due to an insertion of repeat sequences in the A + T-rich region. In addition, the mitogenome of MED has the same gene order as that of New World species, which differs from the gene order of postulated whitefly ancestor [14].

**Table 1:** Mitochondrial genes of MED as determined by DOGMA

**Gene**	**Strand**	**From**	**To**	**Length (bp)**	**Start**	**Stop**
*cox1*	+	1	1537	1537	ATG	T
*tRNA-LeuUUR*	+	1538	1602	65		
*cox2*	+	1603	2266	664	ATA	T
*tRNA-LysAAR*	+	2267	2334	68		
*atp8*	+	2360	2593	234	ATG	TAG
*atp6*	+	2583	3233	651	ATG	TAA
*tRNA-SerUCN*	_	3278	3334	57		
*tRNA-GluGAR*	_	3345	3407	63		
*tRNA-PheUUY*	_	3423	3489	67		
*nd5*	_	3490	5143	1654	ATT	T
*tRNA-HisCAY*	_	5141	5205	65		
*nd4*	_	5206	6498	1293	ATA	TAA
*nd4l*	_	6495	6779	285	ATG	TAA
*tRNA-ThrACN*	+	6781	6858	78		
*tRNA-ProCCN*	_	6847	6908	62		
*nd6*	+	6943	7389	547	ATG	TAA
*cytb*	+	7388	8524	1137	ATG	TAA
*tRNA-SerAGY*	+	8520	8583	64		
*nd1*	_	8582	9517	936	ATT	TAA
*tRNA-LeuCUN*	_	9518	9585	68		
*rrnL*	_	9585	10795	1211		
*tRNA-ValGUN*	_	10796	10863	68		
*tRNA-AspGAY*	_	10867	10938	72		
*tRNA-GlnCAR*	_	10944	11007	64		
*rrnS*	_	11017	11687	671		
*tRNA-AsnAAY*	_	11917	11980	64		
*tRNA-ArgCGN*	_	11981	12049	69		
*tRNA-AlaGCN*	_	12053	12117	65		
*nd3*	_	12123	12476	354	ATG	TAA
*tRNA-GlyGGN*	_	12478	12540	63		
*cox3*	_	12569	13354	786	ATT	TAG
*tRNA-IleAUN*	+	14328	14396	65		
*tRNA-MetAUG*	+	14395	14462	68		
*nd2*	+	14478	15431	954	ATC	TAA
*tRNA-TrpUGG*	+	15430	15497	68		
*tRNA-TyrUAY*	_	15496	15558	63		
*tRNA-CysUGY*	_	15557	15630	74		

### Sequence divergence between mitogenoms of the MED and New World *B. tabaci* species

An earlier study of the whitefly mitogenomes focused on the gene arrangement in the New World species [29]. To understand the divergence and evolution of *B. tabaci* mitogenomes, the differences between MED and New World mitogenomes (GenBank accession number: AY521259) were analyzed in detail. For the 13 PCGs, the overall divergence between MED and New World is 21.30%, which is higher than the divergence of *cox1* sequences (14.9%) which was used to define the *B. tabaci* species limits [12]. The nucleotide sites in PCGs were further classified into nondegenerate (nd) (any nucleotide substitutions produce amino acid change) and fourfold degenerate sites (4d) (no changes cause amino acid replacement). The 4d sites within PCGs are free from selective constraints and can provide data for inferring evolutionary distance [38]. From a total of 10,941 bp of PCGs, 6,150 bp are nd sites, whereas 1,070 bp are 4d sites (Table 2). At 4d sites the overall divergence is 39.95%, whereas the overall divergence at nd sites is only 9.96%. At nd sites, the divergence at non-CpG sites and CpG sites are comparable between mitogenomes of the MED and New World species. However, at 4d sites, the divergence of CpG sites (80.03%) is 2 times of that at the non-CpG sites (37.09%). These results demonstrate that the higher percentage of divergence at the 4d sites is proportional to both the content of CpG sites and the rate of mutation (Table 2).

**Table 2:** Sequence divergence of mitogenome between the invasive MED and New World cryptic species

	**%CpG**	**%GC**	**Loci**	**% mean of difference**	**SE**	**Compared kb**	**Ts/Tv**^**d**^
CDS^a^	2.6	26	13				
All				21.30	0.014	10.94	1.72
No CpG				20.88	0.014	10.66	1.69
CpG				36.21	0.040	0.28	2.38
nd sites^b^	2.83	29.89	13				
All				9.96	0.012	6.15	1.52
No CpG				9.99	0.012	5.98	1.46
CpG				9.87	0.037	0.17	13.00
4d sites^c^	6.36	19.46	13				
All				39.55	0.031	1.07	0.85
No CpG				37.09	0.029	1.00	0.81
CpG				80.03	0.086	0.07	1.14

^a^*CDS* coding sequence.

^b^nd sites: non-degenerative sites.

^c^4d sites: fourfold-degenerate sites where no changes cause any amino acid replacement.

^d^Ts/Tv: ration of transitions (Ts) over transversions (Tv).

**Figure 2:**
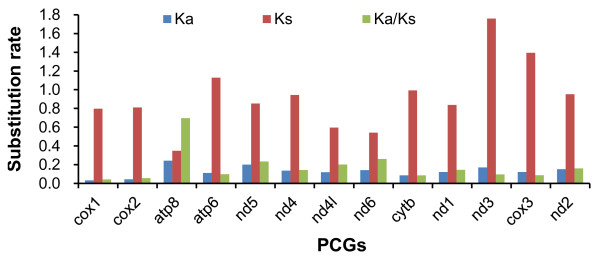
**Evolutionary rates of PCGs between MED and New World mitogenomes.** Ka: nonsynonymous substitution rate;Ks: synonymous substitution rate. The histogram represented the value of Ka, Ks and Ka/Ks for each PCG.

The value of Ka/Ks ratios can be used as an indicator of selective pressure acting on a PCG [39]. To infer the direction and magnitude of natural selection acting on PCGs, the rate of non-synonymous substitutions (Ka), synonymous substitutions (Ks) and the ratio of Ka/Ks were estimated for each PCG between the MED and New World mitogenomes (Figure 2). Among the 13 pairs of PCGs, the Ka/Ks ratio of *atp8* was the highest (0.696), indicating that *atp8* has been evolving under high selection pressure and has the potential value for studying the inherit diversity of different *B. tabaci* populations within a species [40,41]. *Cox1*, *cox2*, *atp6*, *cytb*, *cox3* and *nd3* showed the lowest Ka/Ks values (below 0.1), suggesting that those genes are under high purifying selection.

### Codon usage

**Figure 3:**
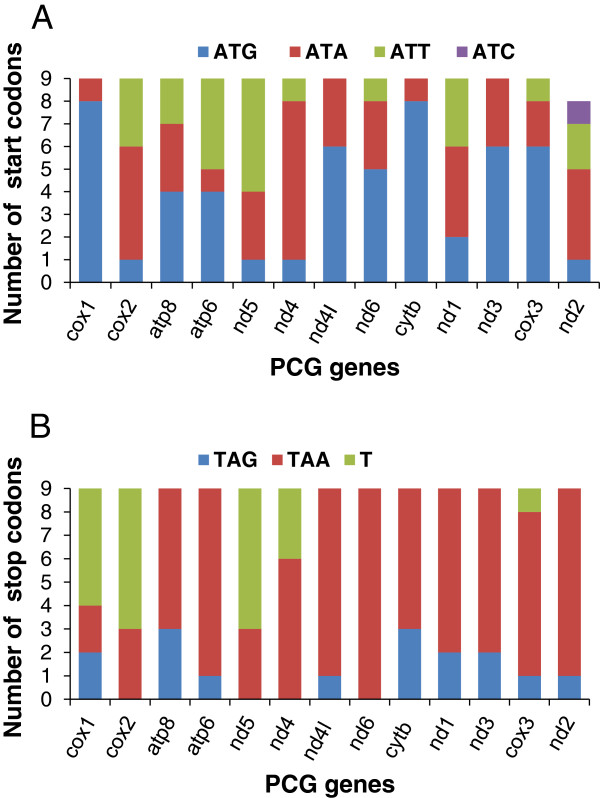
**Usage of start and stop codons in the mitogenomes of nine species.***B.tabaci* (MED), *B. tabaci* (New World), *Tetraleurodes acaciae*, *Neoma*s*kellia andropogonis*, *Aleurochiton aceri*s, *Trialeurodes vaporariorum*, *Aleurodicus dugesii*, *Pachypsylla venusta* and *Schizaphis graminum* respectively. (**A**) Start codons usage of 13 PCGs in nine different species. Because in GenBank the *Trialeurodes vaporariorum nd2* gene is incomplete and does not have the start codon (AY521265), only 8 start codons were shown. (**B**) Stop codons usage of 13 PCGs in nine related species.

The four most frequently used codons were UUU-F (418), UUA-L (394), AUU-I (298) and UAU-Y (262) (Additional file 3) in MED species, while in the New World were UUU-F (404), UUA-L (316), AUU-I (306) and AAU-N (306) (Additional file 4). To compare the usage of start and stop codons in species closely related to *B. tabaci*, nine species (including seven species of whiteflies, 1 psyllid and 1 aphid) within the suborder Sternorrhyncha (Hemiptera) were used [42]. Results show that start codons (Additional file 5) and stop codons (Additional file 6) are used at different frequencies (Figure 3A &3B). Incomplete stop codons have previously been found in insect mitogenomes and are common in metazoans [37]. In the process of post-transcriptional modification, the incomplete stop codon is polyadenylated to complete poly(A) tail and changed T or TA to the TAA stop codon [43]. In the MED mitogenome, complete translation termination codons were used by ten genes, excluding *cox1*, *cox2* and *nd5*, which have incomplete stop codons [24,44-46]. These three genes also used the incomplete T stop codon in the New World species. But in other seven species, incomplete stop codons were also used by different genes (Additional file 6).

### Control regions

**Figure 4:**
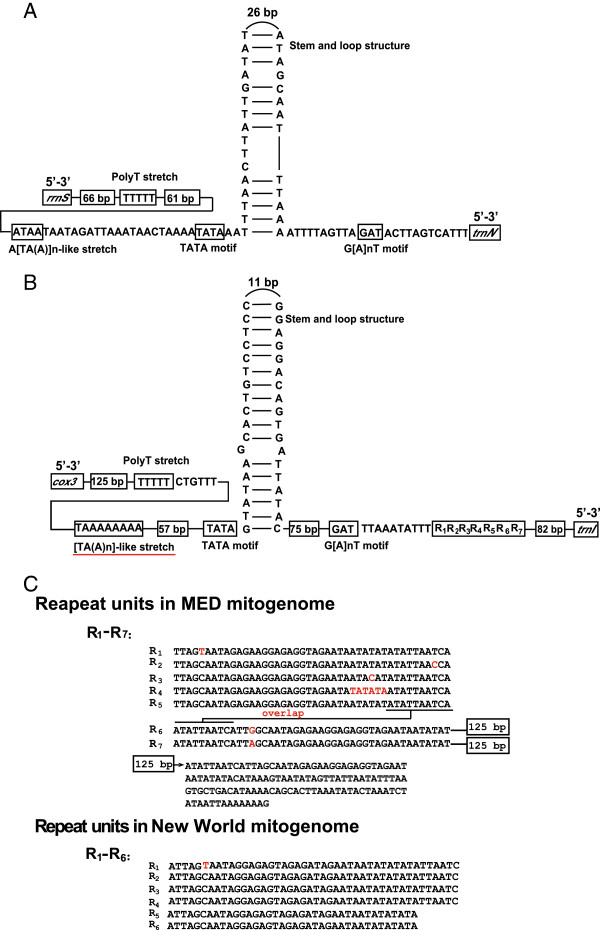
**Predicted transcription motifs for the two control regions of the MED mitogenome.** (**A**) The first control region (CR1) and conserved transcription motifs. (**B**) The second control region (CR2) and conserved transcription motifs. The red line under the [TA(A)n]-like stretch represents the different motif from the New World species. (**C**) Tandem repeat in the second control region of MED and New World mitogenomes. In MED, R_1_-R_7_ represents the tandem repetition in the second region. The nucleotides with red color mean divergence between repeat units and the overlap means the two repeat units share the same 10 nucleotides. The repeat unites of R_1_-R_5_ are 44 bp long and the length of R_6_, R_7_ are 169 bp. The 125 bp represent R_6_ and R_7_ have the same 125 bp repeat nucleotides. In New World species, R_1_-R_6_ represents the six repeat regions in the second region. R_1_-R_4_ repeat unit is 44 bp long and R_5_, R_6_ repeat length is 38 bp.

Using long-PCR, two large non-coding regions were recovered from the MED mitogenome, which was called putative control regions because of the following reasons. First, these two large non-coding regions can form stem-loop structures, which typically associated with the origins of replication/transcription. Second, elements related to transcription and DNA replication were found in both regions. Third, New World *B. tabaci* was shown to have two control regions (one small and one large) [47]. Sequence analyses revealed that the two putative control regions were located in the same region in the mitogenomes of MED and New World species. In both species, one of the putative control regions was located between *rrnS* and *trnN*, while the other located between *cox3* and *trnI* (Figure 1). By analyzing these two areas, elements proposed to be involved in mitogenome replication and gene transcription were found in both control regions (Figure 4). In the first putative control region, both the MED and New World mitogenomes have the same five elements: polyT stretch, A[TA(A)]_n_-like stretch, TATA motif, stem and loop structure and G[A]_n_T motif (Figure 4A). However, in the second putative control region, the sequence of the transcription elements and the tandem repeat regions are different [47]. In the second putative control region of the MED mitogenome, the [TA(A)n]-like stretch differs from the A[TA(A)]n-like stretch in that of New World (Figure 4B). In addition, seven repeat units are found in the second control region of the MED mitogenome. Repeat units of R_1_-R_5_ are 44 bp long and the length of R_6_, R_7_ is 169 bp (Figure 4C). However, the second putative control region of the New World mitogenome contains 6 repeat unites, in which the R_1_-R_4_ repeat units are 44 bp long and the R_5_, R_6_ repeats are 38 bp (Figure 4C).

### Transcript reads mapping to the MED mitogenome

**Figure 5:**
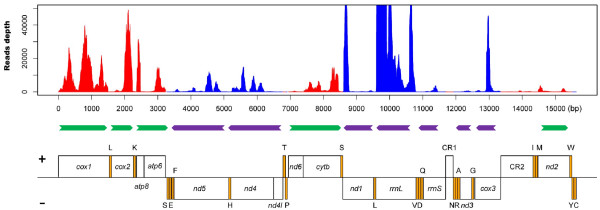
**Depth of raw reads mapped to the MED mitogenome.** The x-axis shows the relative position of mitochondrial genes and the two control regions, and the y-axis represents the number of mapped reads at each nucleotide. Genes coded in the J strand are shown in red and genes coded in the N strand are shown in blue, while non-coding region are shown in gray. The labeled boxes represent PCGs and rRNA genes and the yellow boxes represent tRNAs. The arrows represent the transcription units and their direction of transcription. The bold black line represents the boundary of the J strand and N strand.

NGS technologies have been applied to analyze the gene expression profile of mitochondrial-derived transcripts [21,33]. Previously, we have sequenced the transcriptome of MED and obtained 43 million 75 bp reads [36]. In this study, those sequencing reads were mapped onto the complete MED mitogenome and the numbers of reads per base calculated (read coverage). Based on the mapped reads, we analyzed the reads depth for every base of the MED mitogenome and found that the expression levels of individual mitochondrial genes (13 PCGs and 2 rRNAs) varied greatly from 21 to 236,749 reads per base (Additional file 7). For all coding regions, *rrnL* had the highest expression level, while *nd4l* the lowest. This result is similar to the findings in *Drosophila melanogaster* and the pod borer *Maruca vitrata*[21,33]. The expression level of other PCGs, from highest to lowest, were *cox2*, *cox1*, *atp8*, *nd1*, *cox3*, *cytb*, *atp6*, *nd4*, *nd5*, *nd2*, *rrnS*, *nd3* and *nd6*. Such differences suggest that the expression profile of mitochondrial genes is highly variable (Figure 5) [35].

### Reads mapped to tRNAs and control regions

Reads mapped to tRNA, PCGs and control regions can reveal different features for each of the segments. In the MED mitogenome, the read coverage of tRNAs was relatively low compared to that of PCGs and no reads were recovered for some of tRNAs. In addition, a number of reads were mapped to putative control regions, indicating the existence of non-coding RNA (ncRNA). The expression levels for the first and the second control regions were 0.44 and 90.93, respectively, suggesting that the two putative control regions were transcribed at different levels. However, both functional and comparative studies are needed to examine whether these two putative control regions are real control regions [48,49]. In addition, some reads were also mapped to the intragenic spacer region and the expression level varied (Figure 5). These results suggest that while the control and intervening spacer regions are transcribed, their expression levels are lower than that of PCGs. Interestingly, the same phenomenon has been found in the mitogenomes of mouse, pig and salamander [33,50]. The latest research also revealed that the mammalian mitogenomes encode abundant ncRNAs besides the 37 known mitochondrial genes [51]. Whether these ncRNAs may play a role in post-transcriptional processing or simply reflect polycistronic transcription warrants further investigation.

### Polyadenylation of mitochondrial PCGs

**Figure 6:**
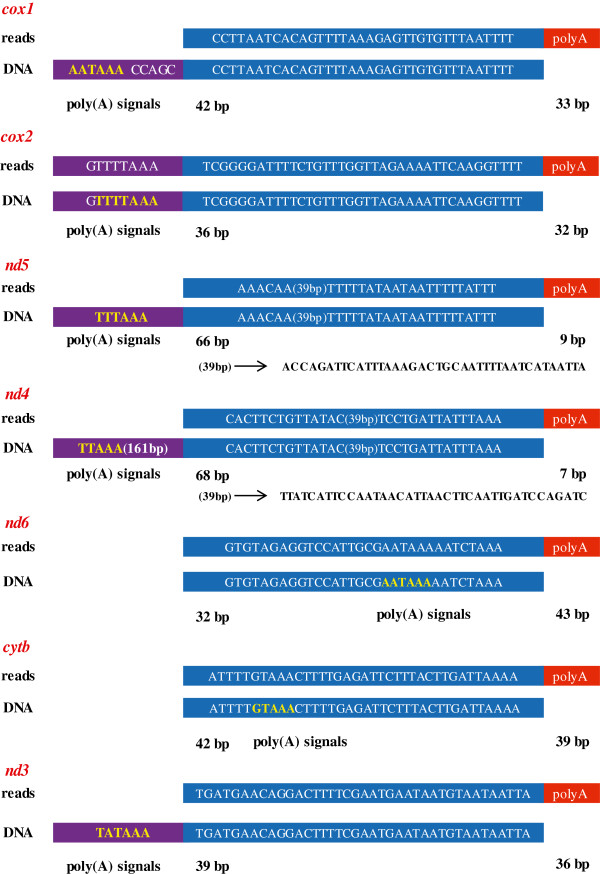
**Alignment of reads with polyA(T) tails to mitochondrial PCGs.** The upper sequence in each group is from the RNA-seq sequencing reads (75 bp). The lower sequence represents the partial gene sequence of PCGs. The possible poly(A) signals are shown in yellow color. The nucleotides highlighted in blue represent the same sequences from reads and PCGs. The nucleotides highlighted in purple boxes represent possible poly(A) signals or sequences in PCGs. The numbers under the purple boxes represent the length of poly(A) stretch.

The polyadenylation of mRNAs plays critical roles in regulating gene expression, complementing coding information and determining gene boundaries [52]. To characterize the polyadenylation of MED mitochondrial genes, sequencing reads mapped to the MED mitogenome and having poly(A) stretches at the 3’ end were analyzed further. Interestingly, a number of reads with poly(A) stretches were mapped at the end or downstream of the stop codons of *cox1*, *cox2*, *nd3*, *nd4*, *nd5*, *nd6* and *cytb* genes (Figure 6). In addition, poly(A) tail was found in the downstream of *lrRNA* and *srRNA* too. Similar to the results found in *Drosophila*[27], the partial stop codon in *cox1*, *cox2* and *nd5* is completed via polyadenylation that changed T to the TAA stop codon. Poly(A) signals are a common feature of eukaryotic PCGs [53]. From the reads mapped to the 3’ end of PCGs, some possible poly(A) signals were recognized (Figure 6). Furthermore, as the sequencing reads are only 75 bp, some poly(A) signals were found in the upstream mitochondrial DNA sequence. All of those poly(A) signals were recognized 8–229 bp upstream of the poly(A) tail. In the 7 PCGs with poly(A) tails, different poly(A) signals were found (Figure 6). These findings confirm that whitefly mitochondrial mRNAs possess poly(A) tail and 3’ proximal poly(A) signals. In addition, these polyadenylation information was employed to annotate the exact termination site of PCGs [27]. Indeed, the location of poly(A) in the 7 PCGs supports the termination site of PCGs predicted via homologous annotation (data not shown).

### MED mitochondrial polycistronic transcripts

Generation of polycistronic transcripts is a distinct feature of many mitogenomes, and the co-transcription of genes may be used for regulation of gene expression [54]. Two genes on the same mature polycistronic transcripts had been reported in the dipteran insects *D. melanogaster*, *D. pseudoobscura* and lepidopteran insect *M. vitrata*[21,33]. To detect possible mature dicistronic transcripts, the assembled MED transcriptome sequences were mapped to the MED mitogenome. Interestingly the *atp6* and *atp8* genes, *nd4l* and *nd4* genes, and *nd6* and *cytb* genes were found in the same contigs respectively (Table 3). The assembled contigs may represent mature transcripts that are generated from tRNA punctuated cleavage of polycistronic transcripts [43,55]. In addition, we can clearly find dip in expression levels associated with the ends of these genes (*atp8-atp6, nd4l-nd4* and *nd6-cytb*) (Figure 5). This is consistent with a mature dicistronic mRNA. By default, if the mitochondrial genes are contiguous within the genome and lack of intervening tRNA, they are transcribed on the same cistrons. Similarly, *atp6* and *atp8*, *nd4l* and *nd4*, and *nd6* and *cytb* genes are contiguous within the mitogenome and also exist in the same contigs.

**Table 3:** The expression profile of contigs transcribed from the MED mitogenome

**Contigs**	**Genes within contig**	**Contig length (bp)**
> comp32_c0_seq1	*atp8*, *atp6*	851
> comp50_c0_seq1	*nd5*	1480
> comp29_c0_seq2	*nd4*	1081
> comp1997_c0_seq1	*nd4*, *nd4l*	557
> comp34_c0_seq1	*nd6*, *cytb*	1636
> comp4_c0_seq1	*nd1*	546
> comp984_c0_seq1	*nd3*	468
> comp6_c0_seq1	*cox3*	881
> comp249_c0_seq1	*nd2*	1077
> comp0_c0_seq1	*rrnL*	674
> comp252_c0_seq2	*rrnS*	784

### Single nucleotide polymorphism (SNP) in the MED mitogenome

SNPs might be useful for discovering genes under selection and the dynamics of these genes in natural populations [56]. Therefore SNPs in the MED mitogenome were investigated using the 43 million Illumina sequencing reads and only 8 SNPs were discovered (Table 4). Among the 8 SNPs, 5 were found in the coding region and 3 were observed in the control region. Interestingly, all the 5 SNPs in the coding region (*cox1*, *cox2*, *nd5*, *cytb*, *nd2*) were C/G to A/T mutation; and only 2 SNPs in the gene of *cox2*, *nd5* lead to the amino acid changes. As the whitefly specimens used for RNA-Seq was derived from a laboratory colony, it is possible few SNPs were identified due to clonality. For practical purposes, field-collected whitefly populations, or species that had undergone different lab selections, regimes should be used to identify SNPs.

**Table 4:** The SNP sites in the complete mitogenome

**Pos**	**Ref**	**Alt**	**Gene_name**	**Strand**	**SNP_position_in_gene**	**Three_base_ref- > three_base_alt**	**aa_ref- > aa_alt**
984	G	A	*cox1*	+	984	ATG- > ATA	M- > M
1873	G	A	*cox2*	+	271	GAA- > AAA	E- > K
5020	C	T	*nd5*	-	124	GGA- > AGA	G- > S
7510	C	T	*cytb*	+	123	GTC- > GTT	V- > V
14675	G	A	*nd2*	+	198	ACG- > ACA	T- > T
14011	A	G	CR region				
14090	G	A	CR region				
14180	A	G	CR region				

Pos: Site position in the complete mitogenome.

Ref: The base on mitochondrial sequence of MED.

Alt: The mutation base.

### Detecting PCG gene expression in different developmental stages

**Figure 7:**
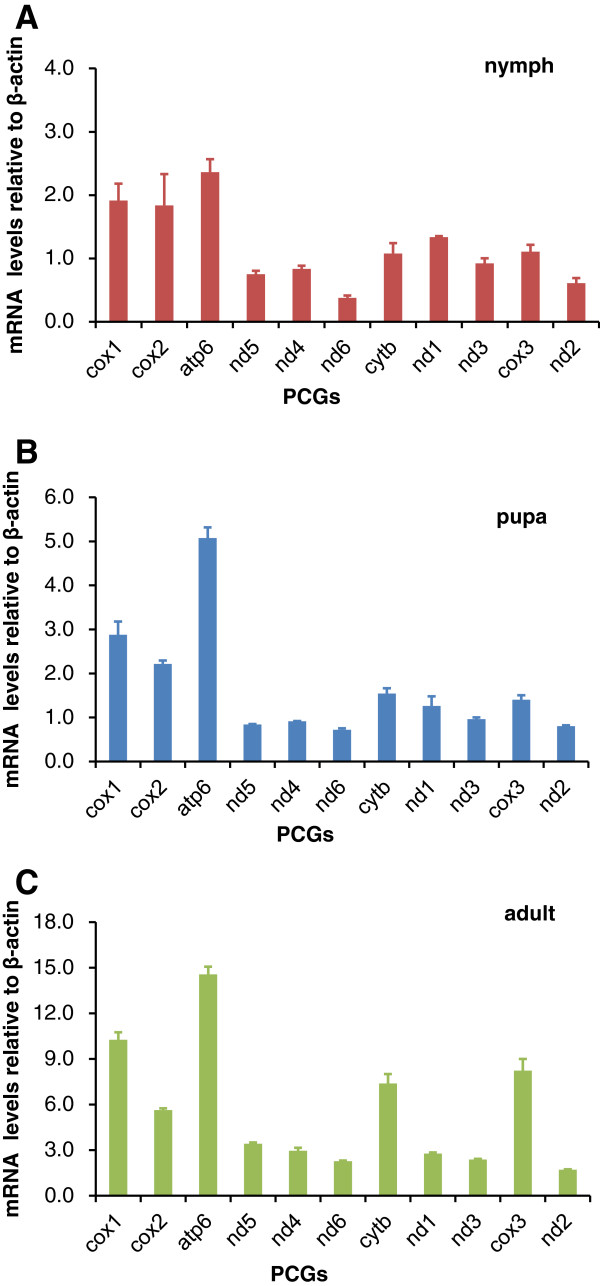
**Expression levels of 11 PCGs in the nymph (A), pupa (B) and adult (C) of MED whiteflies.** Real-Time PCR was used to detect genes expression levels. The x-axis shows the different PCGs, and the y-axis represents the mRNA expression level of different developing stages compared to each control (β-actin).

The mitogenome encodes proteins in the electron transport chain including NADH dehydrogenase (complex I), Cytochrome b (complex III), and Cytochrome c oxidase (complex IV) and ATP synthase (F_0_F_1_-complex). These proteins play important roles in oxidative phosphorylation and ATP generation. Many studies have shown different gene expression profiles of PCGs at different developmental stages [57-60]. However, the expression profile of mitochondrial genes at different developmental stages is poorly understood. Using RT-PCR, the expression level of 11 PCGs was detected in the MED nymphs, pupae, and adults (*nd4l* and *atp8* were excluded due to their short sequence length). Results showed that the expression level of individual mitochondrial genes varied between each developmental stage (Figure 7). We found that the *atp6* gene had the highest expression level in different development stages. At the adult stage, the expression level of the 11 PCGs displayed greater differences than that of the other two stages. In every stage, genes belonging to respiratory complex III and complex IV were expressed at higher levels than genes belonging to complex I. The same phenomenon existed in the early developmental stages of pig tissues [50]. Interestingly, mRNA levels not only differed among genes encoded by separate transcription units, but also showed significant differences among genes located in the same transcription unit (*nd6* and *cytb*). The same phenomena were found in *Drosophila* and may be related to mRNA stability and post-transcriptional mechanisms [21].

## Discussion

Next-generation sequencing is developing rapidly and many datasets have been generated in organisms whose mitochondrial genome is unknown [61]. However, many of the valuable RNA-seq datasets were not analyzed in details. In this study, we have demonstrated that to obtain the mitogenome (at least partially) based on existed RNA-seq data is possible. This strategy can be valuable for the cloning of mitogenomes from other non-model organisms with a sequenced transcriptome. For the *B. tabaci* complex, even though the mitogenome of New World species has been sequenced, we thought that utilizing Illumina sequence reads to obtain the MED mitogenome was more efficient. Because the mitogenomes of New World and MED species are quite diverged (about 20%), some primers directly designed according to the mitochondrial sequence of New World species may not be used to clone MED genes. However, PCR primers can be designed according to the MED Illumina reads mapped to the New World mitogenome with nearly 100% confidence, therefore improve the probability of success (see Additional file 1). As many transcriptomes have been generated from different species, the transcriptome led approach is a useful way to extend existing data. In addition, this method may possibly be a solution or a guide for difficult to sequence mitogenomes.

The mitogenome of MED, which is the type species of the *B. tabaci* complex, shows similarities to the previously published mitogenome of the New World species. Both the mitogenomes have the identical set of genes in the same gene order with two putative control regions (Figure 1). However, the MED genome is slightly longer due to the presence of additional variable repeat sequences in the second control region. Codon usage differs between MED and New World mitogenomes. Overall, the MED mitogenome has 21.30% sequence divergence from that of the New World species, which is higher than the divergence at the *cox1* barcode region (14.9%). This is probably due to the fact that the *cox1* sequences are more constrained than the other 12 PCGs and the presence of divergent noncoding regions (Table 2 and Figure 1). This finding is consistent with the previous claim that mitochondrial genes are susceptible to rapid evolution inferred from higher mutation rates and limited DNA repair mechanisms. The analysis of synonymous and non-synonymous sites of PCGs between MED and New World showed that *atp8* was evolving under high selective pressure (Figure 2), whereas *cox1*, *cox2*, *cox3* and *atp6* had the lowest substitution rates. This finding suggests that *cox1*, *cox2*, *cox3*, and *atp6* may be used for reconstructing evolutionary relationships at the species level, while *atp8* may be suitable for population level phylogenetic analysis.

EST data is important to define gene boundaries, predict processed mitochondrial transcripts and reveal transcription process of mitogenome. Mapping MED RNA-seq data to its mitogenome revealed a number of interesting characteristics about the MED mitogenome, such as gene expression, noncoding RNA, RNA polyadenylation and cistronic transcript. Noncoding RNAs play important roles in the splicing site recognition during the processing of transcripts if they have the ability to form stable stem-loop structures [46,63]. Intergenic noncoding RNAs were found in the MED mitogenome. Previous RNA-seq analyses had revealed that a number of intergenic noncoding RNAs are expressed [64,65] and noncoding RNAs appear to contain functional information [66], including transcription, RNA splicing, editing, translation and turnover [67]. UTRs and intronic regions flanking nuclear genes are critical for regulating its expression, but mitogenome lacks of these regions, indicating that the mitochondrial noncoding RNA may serve as a backup mechanism to coordinate gene expression [21]. Whether noncoding RNAs found in MED mitogenome have similar functions warrants further investigation. From the transcriptome data, *rrnL* had the highest expression level*.* In *Drosophila*, the mitochondrial termination factor mTERF binds just downstream of the 3′ end of the ribosomal gene cluster and is responsible for the higher expression levels of rRNAs [68]. In New World mitogenome, the putative mTERF binding site (ACTAA) should locate in the non-coding DNA between *nd1* and tRNA-Ser, similar to Philaenus (AACTAT) which is the hemipteran and very similar to Lepidoptera [69]. In MED mitogenome, although there is no non-coding region between *nd1* and tRNA-Ser, the same consensus sequence (ACTAA) was discovered at the 3' end of *nd1*. Interestingly, there are instances in beetles of frame shift mutations causing the mTERF site to become part of the coding region despite no changes to the sequence of the recognition site [70]. Therefore, we propose this region as the mTERF binding site in the MED mitogenome. Interestingly, the mTERF domain-containing protein 1-like and domain-containing protein 2-like were found in transctiptome data of MED (data unpublished). This further suggests that the mTERF binding site exists in the MED mitogenome.

In MED, *atp8/6, nd4l/nd4* and *nd6/cytb* genes were found in the same dicistronic transcripts in mature mRNA. Generation of polycistronic transcripts is a common feature of many mitochondria and the co-transcription of genes is likely used for the regulation of gene expression [36]. Previous research has demonstrated that mRNAs that are smaller than ~400 nucleotides interact with 28S subunit of the ribosome less readily than larger mRNAs and for efficient binding, thus a minimum transcript length of ~400 nucleotides is necessary [71,72]. This may partially explain why some mRNAs (i.e. *nd4l/nd4* and *atp8/6*) in the MED mitogenome are dicistronic. In these dicistronic mRNAs, both *nad4l* and *atp8* are shorter than ~400 nucleotides. Therefore, both *nad4l* and *atp8* need to form a dicistronic mRNA with downstream genes to initiate the protein translation efficiently [71]. In dicistronic transcripts, the downstream gene lose the 5' untranslated region, which are capable of forming extensive secondary structures and play important roles in post-transcriptional events [73-75]. In dicistronic transcripts of MED, absence of 5' untranslated regions of *atp6*, *nd4*, *cytb* genes may increase the efficiency of translation, suggesting a different role for the persistence of dicistronic molecules [76]. Interestingly, for the dicistronic *nd6/cytb* and *atp8/atp6* transcripts, poly(A) stretches were found in the 3' end of *nd6* and *atp8* genes. Similarly, in the tricistronic transcript (*atp8/atp6/cox3*) of *Drosophila,* poly(A) stretches were found in the 3' terminus of *atp8* and *atp6*, which suggest the variation in mitochondrial transcript cleavage events may occur in the insects [27,33]. Analyses of mitochondrial transcripts in additional species are needed to reveal the mechanism of the polycistronic processing in mitochondria.

The poly(A) tail has been identified to possibly contribute to translational control [73,74] and mRNA degradation [75]. In this study, PCGs of the MED mitogenome were found to have varying lengths of poly(A) tails and some of the poly(A) tails were critical to generate the UAA termination codon. Previous studies on mRNA polyadenylation concluded that the central sequence motif AAUAAA was essential for mRNA polyadenylation and 3' end formation, but recent studies of EST databases suggest that the frequency of the motif appeared low [76]. In our study, different possible poly(A) signals were also found in eight PCGs of the MED mitogenome.

## Conclusions

In summary, the mitogenome of the invasive *B. tabaci* MED species contains the same gene rearrangement as that of the New World species. Using transcriptome data, the expression profile and the termination location of some genes were determined. In addition, polyadenylation, polycistronic transcripts and SNPs were discovered in *B. tabaci* mitogenome for the first time. The results presented here also demonstrate that utilizing RNA-seq data to analyze gene expression characteristics of mitogenome is practical. The MED and New World mitogenomes are interesting but the real utility of the sequence data comes from a comparative approach and it is our recommendation to sequence all of the mitogenomes for the species. With the inclusion of additional mitogenomes, patterns of mitochondrial gene expression and differences of energy usage in invasive and indigenous species could be tested.

## Methods

### Mitogenome sequence mapping and assembly

The complete mitogenome sequence of the New World species of *B. tabaci* was downloaded from GenBank: AY 521259 and was used as reference for alignment. MED transcriptome reads were directly mapped onto the New World mitogenome using Blastn [77]. As the mitogenome of New World and MED differ, when reads of MED were mapped to the New World mitogenome two mismatches were allowed. Then, reads mapped to the New World mitogenome were collected and assembled. Based on the position and sequence information of the assembled contigs, PCR primers were designed to complete the mitogenome sequence of MED (Additional file 2).

### PCR amplification, cloning and sequencing

The method of obtaining MED whitefly DNA samples was described in Wang et al. [78]. Total genomic DNA of multiple individuals was isolated using the DNeasy animal tissue kit (Qiagen, Germany) following the manufacturer’s protocol. PCR was carried out in an S1000 Thermal Cycler (Bio-Rad). A 25 μL PCR reaction contained 0.5 μL 10 μmol primers, 2.5 μL 10 × PCR buffer, 2.0 μL 10 mM dNTP, 0.4 μL LA Taq polymerase (Takara, Japan). Short PCRs (<2.0 kb) were carried out using *Taq* DNA polymerase (Takara, Japan) with the following PCR conditions: 95°C for 2 min, followed by 35 cycles of 96°C for 30 s, 44-52°C for 30 s, 72°C for 3 min, as well as a final cycle of 72°C for 10 min. Long PCRs were carried out using LA *Taq* DNA polymerase with the following PCR conditions: 95°C for 2 min, followed by 35 cycles of 96°C for 30 s, 48-56°C for 30 s, 72°C for 3 min and a final cycle of 72°C for 10 min. PCR fragments were purified and ligated into the pGEM-T Easy Vector (Promega, USA) and sequenced in both directions using the ABI BigDye 3.1 at GenScript (Nanjing, China).

### Annotation of the MED mitogenome

DOGMA [79] was used to annotate the PCGs, rRNA genes of MED mitochondrial DNA. tRNAs were identified using tRNAscan-SE (invertebrate mitochondrial genetic code and ‘mito/chloroplast’ source) [80]. tRNA genes that could not be identified using tRNAscan-SE, sequences were aligned with published Aleyrodidae mitochondrial sequences (see below for species). BioEdit was used to calculate A/T content and also to translate DNA into amino acids [81]. AT and GC skews were calculated by (A-T)/(A+T) and (G-C)/(G+C) respectively [29]. The complete mitogenome of MED was deposited in GenBank: JQ906700.

### Comparison of mitogenomes

The numbers of synonymous substitutions (Ks) and non-synonymous substitutions (Ka) for each gene were calculated with the software of DnaSP Version 5.10.01 [82,83]. For the sequence divergence analyses, pair-wise alignments were generated for all the 13 PCGs orthologous gene pairs based on protein sequences and DNA sequences using the MegaBlast algorithm. According to the mitochondrial codons, the divergence was determined for the contexts of nd, 4d, CpG and non-CpG sites [84]. The ratio of transitions over transversions (Ts/Tv) was caculated for the all coding region as well. The mitogenome sequences of *Tetraleurodes acaciae* (AY521626), *Neoma*s*kellia andropogonis* (AY572539), *Aleurochiton aceris* (AY572538), *Trialeurodes vaporariorum* (AY521265), *Aleurodicus dugesii* (AY521251), *Pachypsylla venusta* (AY278317) and *Schizaphis graminum* (AY531391) were obtained from GenBank. The MED and New World partial *cox1* sequences were extracted from GenBank AM176574 and AY057133, respectively.

### Mapping reads to the MED mitogenome, expression profiling and SNP analysis

TopHat (Version:1.4.1) was used to align the MED NGS reads with the MED mitogenome with the following parameters: -g1 -r 200 –mate-std-dev20 -I 10000 [85]. The numbers of mapped reads for each gene were summed and divided by gene length to calculate the expression level of each mitochondrial gene. To detect polyadenylation, reads containing more than eight continuous A or T from the transcript data were aligned to the 13 mitochondrial genes using Blast. Reads that hit at or downstream of stop codons of PCGs were selected. Based on the mapping results, SAMTools (V0.1.13) were used to discovery the possible SNP sites with depth of at least 10 reads [86]. The analyses of amino acid mutation at PCGs and intergenic region were performed by a custom-written algorithm (available upon request) using mitochondrial codons.

### Verification of dicistronic transcripts

The method of obtaining RNA from MED females had been described [36]. RNA was treated with DNase and 1st Strand cDNA was synthesized following the protocol of PrimeScript II 1st Strand cDNA Synthesis Kit (Takara). Three pairs of primers for the three dicistronic transcripts were designed respectively. PCR were performed with cDNA as template.

### qPCR analysis of PCG expression

Total RNA was extracted from the three MED samples (nymph, pupa and adult) using SV total RNA isolation system (Promega) following the manufacturer’s protocol [87]. The nymph sample include first- to third-instars. The SYBR® Prime Script™ RT-PCR Kit II (Takara) was used to synthesize cDNA and then qPCR was used to detect the expression of 11 mitochondrial genes. The ABI PRISM 7500 Fast Real-Time PCR System (Applied Biosystems) with SYBR-Green detection was employed to perform qPCRs. For normalization, β-actin was selected as the endogenous control. Every gene was analyzed three times and the relative expression levels were calculated by the 2^-△△^Ct method. As an endogenous control, the expression of β-actin was measured in parallel [88].

## Abbreviations

cox1: Cytochrome oxidase, subunit I; cox2: Cytochrome oxidase, subunit II; atp8: ATP synthase, subunit 8; atp6: ATP synthase, subunit 6; nd5: NADH dehydrogenase, subunit 5; nd4: NADH dehydrogenase, subunit 4; nd4l: NADH dehydrogenase, subunit 4L; nd6: NADH dehydrogenase, subunit 6; cytb: Cytochrome b; nd1: NADH dehydrogenase, subunit 1; nd2: NADH dehydrogenase, subunit 2; nd3: NADH dehydrogenase, subunit 3; cox3: cytochrome oxidase, subunit III; nd2: NADH dehydrogenase, subunit 2; rrnS: Small subunit of mitochondrial ribosomal DNA; rrnL: Large subunit of mitochondrial ribosomal DNA.

## Competing interests

The author(s) declare that they have no competing interests.

## Authors’ contributions

HLW, XWW and SSL conceived and designed the experimental plan. HLW and JY preformed the experiments. HLW, JY, LMB, QYZ, QL, XWW and SSL analyzed interpreted the sequence data. HLW, JY, LMB, XWW and SSL drafted the manuscript. All authors read and approved the final manuscript.

## Supplementary Material

Additional file 1 **A diagram about our strategy to obtain the MED mitogenome sequence.** The picture indicates how to get the complete MED mitogenome based on the transcriptome reads mapped to the New World mitogenome. Click here for file

Additional file 2 Primers used to get the complete MED mitogenome. Click here for file

Additional file 3 **Relative synonymous codon usage of the MED species.** The frequency of synonymous codon (include stop codons) usage were shown. Click here for file

Additional file 4 **Relative synonymous codon usage of the New Worlds species.** The frequency of synonymous codon (include stop codons) usage were shown. Click here for file

Additional file 5 **Start codons usage in nine different species.** Start condons of 13 mitochondrial PCGs were shown. The 9 species are *Bemisia tabaci* (MED), *B. tabaci* (New World), *Tetraleurodes acaciae*, *Neoma*s*kellia andropogonis*, *Aleurochiton aceri*s, *Trialeurodes vaporariorum*, *Aleurodicus dugesii*, *Pachypsylla venusta* and *Schizaphis graminum*. Click here for file

Additional file 6 **Stop codon usage in 9 different species.** Stop codons of 13 mitochondrial PCGs were shown. The 9 species are *Bemisia tabaci* (MED), *B. tabaci* (New World), *Tetraleurodes acaciae*, *Neoma*s*kellia andropogonis*, *Aleurochiton aceri*s, *Trialeurodes vaporariorum*, *Aleurodicus dugesii*, *Pachypsylla venusta* and *Schizaphis graminum.* Click here for file

Additional file 7 **The expression level of PCGs.** By calculating the number of mapped reads, the expression level of 13 mitochondrial PCGs were revealed. Click here for file
