# Supplementary figures and images for: The characteristics and expression profiles of the mitochondrial genome for the Mediterranean species of the Bemisia tabaci complex
Source: BMC Genomics. 2013 Jun 17;14:401. doi: 10.1186/1471-2164-14-401 (PMC3691742; doi:10.1186/1471-2164-14-401)

# Additional file 1

## The New World mitogenome

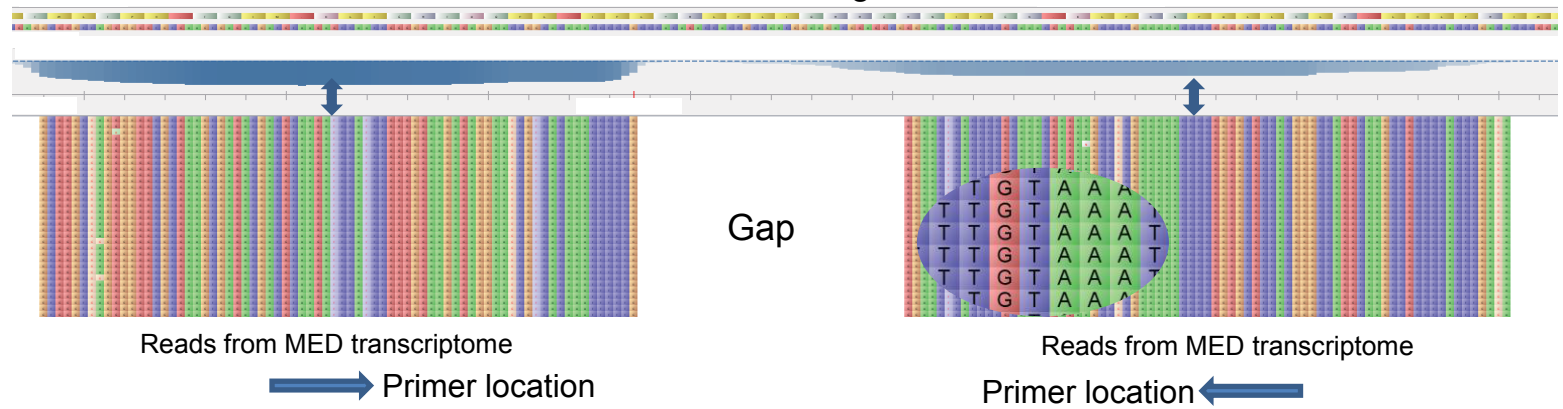

Supplement: Additional file 1 — A diagram about our strategy to obtain the MED mitogenome sequence. The picture indicates how to get the complete MED mitogenome based on the transcriptome reads mapped to the New World mitogenome. [file 1471-2164-14-401-S1.pdf]
