# Supplementary material for: The characteristics and expression profiles of the mitochondrial genome for the Mediterranean species of the Bemisia tabaci complex
Source: BMC Genomics. 2013 Jun 17;14:401. doi: 10.1186/1471-2164-14-401 (PMC3691742; doi:10.1186/1471-2164-14-401)
Supplement: Additional file 2 — Primers used to get the complete MED mitogenome. [file 1471-2164-14-401-S2.doc]

# Additional file 2: Primers used to get the complete MED mitogenome.

| Primer pair | Forward | Reverse |
| --- | --- | --- |
| gap1 | TGGTGTTTGGAGAGGATTAATTGGAACTTC | TAAAATAGGATCCCCTCCTCCTAGAGGAT |
| gap2 | ATCCTCTAGGAGGAGGGGATCCTATTTTA | ATTGGTTTAGCCGACCAGGAACAGAAT |
| gap3 | ATTCTGTTCCTGGTCGGCTAAACCAAT | CTGCGGAATTAAGCTAAACTAGCCTTCAT |
| gap4 | ATGAAGGCTAGTTTAGCTTAATTCCGCAG | CGAATTAACCAGCCATTATTAACATCTCGC |
| gap5 | GCGAGATGTTAATAATGGCTGGTTAATTCG | GCTTAATAATTAACGTCTTCGTGCCTTCG |
| gap6,7 | CGAAGGCACGAAGACGTTAATTATTAAGC | ACCCTCTCTAGGGGTTGCCCTATAAT |
| gap8 | ATTATAGGGCAACCCCTAGAGAGGGT | GAAGTTCCAATTAATCCTCTCCAAACACCA |
| 13603-14095 | CCACAATAGGAGGACAGTGATTATAC | TCTATAGTTGAAATATGAGTCCAATAGCCTTAG |
| 14000-15000 | TGCTGATTTAGGGTTATGAGATGGAC | CATTTTACTCACCTACTAAATACCAATATGAC |
