# Supplementary material for: The characteristics and expression profiles of the mitochondrial genome for the Mediterranean species of the Bemisia tabaci complex
Source: BMC Genomics. 2013 Jun 17;14:401. doi: 10.1186/1471-2164-14-401 (PMC3691742; doi:10.1186/1471-2164-14-401)
Supplement: Additional file 3 — Relative synonymous codon usage of the MED species. The frequency of synonymous codon (include stop codons) usage were shown. [file 1471-2164-14-401-S3.doc]

**Additional file 3: Relative synonymous codon usage of the MED mitogenome.**

|  |  |  |  |  |  |  |  |
| --- | --- | --- | --- | --- | --- | --- | --- |
| UUU-F | 418(1.62) | UCU-S | 115(1.96) | UAU-Y | 262(1.68) | UGU-C | 70(1.52) |
| UUC-F | 99(0.38) | UCC-S | 32(0.54) | UAC-Y | 50(0.32) | UGC-C | 22(0.48) |
| UUA-L | 394(2.93) | UCA-S | 90(1.53) | UAA-* | 290(1.55) | UGA-W | 92(1.23) |
| UUG-L | 117(0.87) | UCG-S | 14(0.24) | UAG-* | 83(0.45) | UGG-W | 58(0.77) |
| CUU-L | 95(0.71) | CCU-P | 36(1.69) | CAU-H | 68(1.58) | CGU-R | 13(1.11) |
| CUC-L | 31(0.23) | CCC-P | 19(0.89) | CAC-H | 18(0.42) | CGC-R | 8(0.68) |
| CUA-L | 117(0.87) | CCA-P | 23(1.08) | CAA-Q | 69(1.42) | CGA-R | 18(1.53) |
| CUG-L | 53(0.39) | CCG-P | 7(0.33) | CAG-Q | 28(0.58) | CGG-R | 8(0.68) |
| AUU-I | 298(1.51) | ACU-T | 87(2.04) | AAU-N | 239(1.65) | AGU-S | 61(1.04) |
| AUC-I | 97(0.49) | ACC-T | 22(0.51) | AAC-N | 51(0.35) | AGC-S | 33(0.56) |
| AUA-M | 194(1.41) | ACA-T | 53(1.24) | AAA-K | 220(1.51) | AGA-S | 90(1.53) |
| AUG-M | 81(0.59) | ACG-T | 9(0.21) | AAG-K | 72(0.49) | AGG-S | 35(0.60) |
| GUU-V | 126(1.98) | GCU-A | 44(2.15) | GAU-D | 94(1.66) | GGU-G | 68(1.49) |
| GUC-V | 21(0.33) | GCC-A | 7(0.34) | GAC-D | 19(0.34) | GGC-G | 24(0.52) |
| GUA-V | 73(1.15) | GCA-A | 24(1.17) | GAA-E | 82(1.39) | GGA-G | 52(1.14) |
| GUG-V | 34(0.54) | GCG-A | 7(0.34) | GAG-E | 36(0.61) | GGG-G | 39(0.85) |

* represent stop codons
