# Supplementary material for: The characteristics and expression profiles of the mitochondrial genome for the Mediterranean species of the Bemisia tabaci complex
Source: BMC Genomics. 2013 Jun 17;14:401. doi: 10.1186/1471-2164-14-401 (PMC3691742; doi:10.1186/1471-2164-14-401)
Supplement: Additional file 4 — Relative synonymous codon usage of the New Worlds species. The frequency of synonymous codon (include stop codons) usage were shown. [file 1471-2164-14-401-S4.doc]

**Additional file 4：Relative synonymous codon usage of the New World mitogenome**

|  |  |  |  |  |  |  |  |
| --- | --- | --- | --- | --- | --- | --- | --- |
| UUU-F | 404(1.57) | UCU-S | 142(1.98) | UAU-Y | 316(1.70) | UGU-C | 115(1.61) |
| UUC-F | 110(0.43) | UCC-S | 40 (0.56) | UAC-Y | 56(0.30) | UGC-C | 28 (0.39) |
| UUA-L | 289(2.96) | UCA-S | 78 (1.09) | UAA-* | 263(1.75) | UGA-W | 86 (0.57) |
| UUG-L | 86(0.88) | UCG-S | 18 (0.25) | UAG-* | 102(0.68) | UGG-W | 62 (1.00) |
| CUU-L | 104(1.06) | CCU-P | 45 (1.96) | CAU-H | 73(1.51) | CGU-R | 15 (0.53) |
| CUC-L | 20(0.20) | CCC-P | 13 (0.57) | CAC-H | 24(0.49) | CGC-R | 3 (0.11) |
| CUA-L | 54(0.55) | CCA-P | 28 (1.22) | CAA-Q | 56(1.45) | CGA-R | 14 (0.50) |
| CUG-L | 33(0.34) | CCG-P | 6 (0.26) | CAG-Q | 21(0.55) | CGG-R | 7 (0.25) |
| AUU-I | 306(1.59) | ACU-T | 82 (2.09) | AAU-N | 306(1.65) | AGU-S | 113 (1.57) |
| AUC-I | 87(0.45) | ACC-T | 24 (0.61) | AAC-N | 66(0.35) | AGC-S | 40 (0.56) |
| AUA-I | 186(0.96) | ACA-T | 38 (0.97) | AAA-K | 228(1.55) | AGA-R | 90 (3.20) |
| AUG-M | 51(1.00) | ACG-T | 13 (0.33) | AAG-K | 66(0.45) | AGG-R | 40 (1.42) |
| GUU-V | 129(2.12) | GCU-A | 41 (2.45) | GAU-D | 91(1.61) | GGU-G | 66 (1.68) |
| GUC-V | 30(0.49) | GCC-A | 8 (0.41) | GAC-D | 22(0.39) | GGC-G | 21 (0.54) |
| GUA-V | 60(0.99) | GCA-A | 13 (0.78) | GAA-E | 51(1.28) | GGA-G | 38 (0.97) |
| GUG-V | 24(0.40) | GCG-A | 5 (0.30) | GAG-E | 29(0.73) | GGG-G | 32 (0.82) |

* represent stop codons
