# Supplementary material for: The characteristics and expression profiles of the mitochondrial genome for the Mediterranean species of the Bemisia tabaci complex
Source: BMC Genomics. 2013 Jun 17;14:401. doi: 10.1186/1471-2164-14-401 (PMC3691742; doi:10.1186/1471-2164-14-401)
Supplement: Additional file 5 — Start codons usage in nine different species. Start condons of 13 mitochondrial PCGs were shown. The 9 species are Bemisia tabaci (MED), B. tabaci (New World), Tetraleurodes acaciae, Neomaskellia andropogonis, Aleurochiton aceris, Trialeurodes vaporariorum, Aleurodicus dugesii, Pachypsylla venusta and Schizaphis graminum. [file 1471-2164-14-401-S5.doc]

**Additional file 5: Start codon usage in 9 different species.**

| Start codon | 1* | 2 | 3 | 4 | 5 | 6 | 7 | 8 | 9 |
| --- | --- | --- | --- | --- | --- | --- | --- | --- | --- |
| *cox1* | ATG | ATG | ATG | ATG | ATG | ATG | ATG | ATG | ATA |
| *cox2* | ATA | ATA | ATA | ATA | ATT | ATT | ATG | ATT | ATA |
| *atp8* | ATG | ATG | ATT | ATG | ATA | ATA | ATT | ATG | ATA |
| *atp6* | ATG | ATG | ATT | ATT | ATG | ATT | ATA | ATG | ATT |
| *nd5* | ATT | ATT | ATA | ATT | ATA | ATT | ATT | ATG | ATA |
| *nd4* | ATA | ATA | ATA | ATA | ATT | ATA | ATA | ATG | ATA |
| *nd4l* | ATG | ATG | ATA | ATG | ATG | ATG | ATA | ATA | ATA |
| *nd6* | ATG | ATG | ATG | ATG | ATG | ATA | ATA | ATA | ATT |
| *cytb* | ATG | ATG | ATG | ATG | ATG | ATG | ATG | ATA | ATG |
| *nd1* | ATT | ATG | ATA | ATG | ATA | ATA | ATA | ATT | ATT |
| *nd3* | ATG | ATG | ATG | ATG | ATG | ATG | ATA | ATA | ATA |
| *cox3* | ATT | ATG | ATA | ATG | ATG | ATA | ATG | ATG | ATG |
| *nd2* | ATC | ATA | ATT | ATA | ATT |  | ATA | ATG | ATA |

*1-9 represent the *Bemisia tabaci* (MED), the *Bemisia tabaci* (New World), *Tetraleurodes acaciae*,*Neoma*s*kellia andropogonis*,*Aleurochiton aceris***,** *Trialeurodes vaporariorum*,*Aleurodicus dugesii*,*Pachypsylla venusta* and*Schizaphis graminum* respectively.
