# Supplementary material for: The characteristics and expression profiles of the mitochondrial genome for the Mediterranean species of the Bemisia tabaci complex
Source: BMC Genomics. 2013 Jun 17;14:401. doi: 10.1186/1471-2164-14-401 (PMC3691742; doi:10.1186/1471-2164-14-401)
Supplement: Additional file 6 — Stop codon usage in 9 different species. Stop codons of 13 mitochondrial PCGs were shown. The 9 species are Bemisia tabaci (MED), B. tabaci (New World), Tetraleurodes acaciae, Neomaskellia andropogonis, Aleurochiton aceris, Trialeurodes vaporariorum, Aleurodicus dugesii, Pachypsylla venusta and Schizaphis graminum. [file 1471-2164-14-401-S6.doc]

**Additional file 6: Stop codon usage in 9 different species.**

| Stop codon | 1* | 2* | 3* | 4* | 5* | 6* | 7* | 8* | 9* |
| --- | --- | --- | --- | --- | --- | --- | --- | --- | --- |
| *cox1* | T | T | TAA | TAG | T | TAG | TAA | T | T |
| *cox2* | T | T | T | TAA | TAA | T | T | T | TAA |
| *atp8* | TAG | TAG | TAA | TAA | TAG | TAA | TAA | TAA | TAA |
| *atp6* | TAA | TAA | TAA | TAG | TAA | TAA | TAA | TAA | TAA |
| *nd5* | T | T | T | T | T | TAA | TAA | T | TAA |
| *nd4* | TAA | TAA | T | TAA | TAA | TAA | TAA | T | T |
| *nd4l* | TAA | TAA | TAA | TAA | TAA | TAA | TAA | TAG | TAA |
| *nd6* | TAA | TAA | TAA | TAA | TAA | TAA | TAA | TAA | TAA |
| *cytb* | TAA | TAA | TAA | TAA | TAA | TAG | TAA | TAG | TAG |
| *nd1* | TAA | TAG | TAA | TAG | TAA | TAA | TAA | TAA | TAA |
| *nd3* | TAA | TAA | TAG | TAA | TAG | TAA | TAA | TAA | TAA |
| *cox3* | TAG | TAA | TAA | TAA | TAA | TAA | TAA | T | TAA |
| *nd2* | TAA | TAA | TAA | TAA | TAA | TAG | TAA | TAA | TAA |

*1-9 represent the *Bemisia tabaci* (MED), the *Bemisia tabaci* (New World), *Tetraleurodes acaciae*,*Neoma*s*kellia andropogonis*,*Aleurochiton aceris*, *Trialeurodes vaporariorum*,*Aleurodicus dugesii*,*Pachypsylla venusta* and*Schizaphis graminum* respectively.
