# Supplementary material for: The characteristics and expression profiles of the mitochondrial genome for the Mediterranean species of the Bemisia tabaci complex
Source: BMC Genomics. 2013 Jun 17;14:401. doi: 10.1186/1471-2164-14-401 (PMC3691742; doi:10.1186/1471-2164-14-401)
Supplement: Additional file 7 — The expression level of PCGs. By calculating the number of mapped reads, the expression level of 13 mitochondrial PCGs were revealed. [file 1471-2164-14-401-S7.doc]

**Additional file 7: The expression level by calculating the transcriptome reads mapped to the MED mitogenome.**

| PCGs and two rRNAs | The value of expression level |
| --- | --- |
| *cox1* | 9910 |
| *cox2* | 11121 |
| *atp8* | 9736 |
| *atp6* | 3202 |
| *nd5* | 1573 |
| *nd4* | 2661 |
| *nd4l* | 21 |
| *nd6* | 103 |
| *cytb* | 3590 |
| *nd1* | 6567 |
| *nd3* | 282 |
| *cox3* | 5102 |
| *nd2* | 607 |
| *rrnL* | 236749 |
| *rrnS* | 534 |
| *CR1* | 0.44 |
| *CR2* | 90.93 |

The value of expression level of every gene was calculated by adding up the number of bases of mapped reads for each gene and then divided by the gene length.
